# IPred - integrating ab initio and evidence based gene predictions to improve prediction accuracy

**DOI:** 10.1186/s12864-015-1315-9

**Published:** 2015-02-26

**Authors:** Franziska Zickmann, Bernhard Y Renard

**Affiliations:** Research Group Bioinformatics (NG4), Robert Koch-Institute, Berlin, Germany

**Keywords:** Gene prediction, Gene finder combination, Genome annotation, RNA-Seq integration

## Abstract

**Background:**

Gene prediction is a challenging but crucial part in most genome analysis pipelines. Various methods have evolved that predict genes *ab initio* on reference sequences or evidence based with the help of additional information, such as RNA-Seq reads or EST libraries. However, none of these strategies is bias-free and one method alone does not necessarily provide a complete set of accurate predictions.

**Results:**

We present **IPred** (**I**ntegrative gene **Pred**iction), a method to integrate *ab initio* and evidence based gene identifications to complement the advantages of different prediction strategies. IPred builds on the output of gene finders and generates a new combined set of gene identifications, representing the integrated evidence of the single method predictions.

**Conclusion:**

We evaluate IPred in simulations and real data experiments on *Escherichia Coli* and human data. We show that IPred improves the prediction accuracy in comparison to single method predictions and to existing methods for prediction combination.

**Electronic supplementary material:**

The online version of this article (doi:10.1186/s12864-015-1315-9) contains supplementary material, which is available to authorized users.

## Background

Predicting the genes encoded in the genome of an organism is a crucial part of most pipelines for genomic analysis. Driven by the ongoing advances of high-throughput next-generation sequencing techniques, more and more genome sequences are available that need to be annotated to understand the basis and function of processes in a cell.

To allow accurate predictions, several methods for gene identification have evolved, which can be categorized as *ab initio* and evidence based (including comparative) gene finders. *ab initio* approaches (for instance [1,2]) predict genes exclusively depending on the target sequence and perform identifications based on training data and strategies such as Hidden Markov models [3].

In contrast, evidence based methods compare the sequence of interest to available reference annotations or include external evidence in their prediction, for example EST libraries or RNA-Seq information [4-6].

*ab initio* methods are very sensitive, but their accuracy strongly depends on the quality of the training data set [3]. Further, they have the disadvantage (i) of providing no information on whether the genes are indeed expressed under a certain condition or not, and (ii) of missing or incorrectly predicting genes that differ from the considered standard codon scheme [7]. These criteria are met by evidence based approaches that report genes based on observed evidence and are therefore more robust to non-standard coding schemes. However, these methods are prone to noise in the external information and to incomplete or contradicting evidence [8].

Hybrid approaches, such as AUGUSTUS [9] and JIGSAW [10], combine *ab initio* predictions with other evidence. This allows a more accurate verification of predicted genes, although also hybrid approaches are limited for instance by incomplete evidence or insufficient training data.

Combining the output of different gene prediction strategies allows complementing the strengths of single method predictions to obtain the sensitivity of *ab initio* approaches, while incorporating other evidence to complete and verify identifications, as for example shown in [11] and [12]. Up to now, to the best of our knowledge, approaches combining predictions treat all identifications independently of their prediction strategy and predominantly introduce weighting schemes to score different predictions. Amongst others, these methods include [7,13-17]. Hence, the full complementary potential of the combination of different prediction strategies is not fully tapped. Further, current methods often focus on the integration of a specific set of gene finders [18].

Therefore, in this article we present IPred (Integrative gene Prediction), an automated pipeline to process the output of various gene finders (not focused on certain methods) and merge *ab initio* and evidence based predictions to obtain a new combined set of gene identifications, representing the integrated information of the single method predictions. In particular, IPred is independent of the evidence used to assist gene predictions. It incorporates methods from the full plethora of evidence sources, for instance from EST libraries, protein alignments, sequence comparison, or from increasingly popular RNA-Seq runs. IPred was designed with a focus on combining different prediction strategies to complement their advantages and counterbalance weaknesses of one prediction class with information from another. True positive identifications, for instance highly conserved genes, are likely to be present throughout different evidence information, whereas false positive identifications are not expected to be simultaneously present in the majority of prediction results and can thus be filtered out.

IPred is a flexible and robust method that in contrast to other methods works independently from weighting schemes and does not require any a priori knowledge.

In case a reference annotation is available, all predictions can be automatically evaluated using the framework provided by Cuffcompare [19].

IPred provides its own GUI for easy usability, but can also be applied from the command line. We demonstrate the benefit of our method in two simulations and in two real data experiments based on *Escherichia Coli* and human data.

## Implementation

IPred accepts prediction output files in the commonly used GTF annotation format and provides converter scripts for a range of further file formats, for example the AUGUSTUS GFF format or the Glimmer3 Predict format. The interpretation of GTF format styles can differ among methods. We decided to work based on the GTF format used by the Cufflinks/Cuffcompare suite [19] because we use Cuffcompare in the IPred pipeline (see description below).

Hence, in the following the name *gene* describes a locus on a genome that is expressed to an amino acid sequence. In eukaryotes, a gene can be expressed in more than one *transcript* sequence, also referred to as *alternative isoforms*. Further, the transcribed part of a gene including coding sequence (CDS) and potentially also untranslated sequence (UTR) is called *exon*.

When providing the output of gene finders, the user needs to categorize the different outputs into either *ab initio* or evidence based (including comparative method based) predictions since IPred was in particular designed for combining complementary strategies.

Also hybrid prediction methods and the result of annotation pipelines can be incorporated into IPred. For instance, if a hybrid method is *ab initio* in its nature, it should be specified as *ab initio*. When evidence has been integrated in the annotation pipeline, the result can be specified as evidence based.

Note that it is not recommended to combine *ab initio* with *ab initio* methods since the underlying information, i.e. training sets or employed statistical model, might be very similar and thus could bias the combination of predictions.

However, if an integration of two *ab initio* predictions is desired, one method can be classified as evidence based. Here, it is necessary to keep in mind that potential novel genes that are predicted by the *ab initio* method (that is classified as evidence based) are genes that are not verified by external evidence.

Based on the categorization of each method, IPred first processes the loci of the predicted genes separately and then combines the loci of *ab initio* and evidence based methods. IPred proceeds through the predicted *ab initio* loci (also called “leading" loci) and tests if an evidence based prediction supports this identification. During this process the supported *ab initio* predictions are categorized into genes that perfectly overlap with at least one evidence based prediction and minor supported predictions that have only partial overlap. Note that IPred per default accepts an overlap as a supporting overlap only if it is greater than a threshold of 80% of the length of the original *ab initio* prediction (calculated as the sum of the number of nucleotides of its exons). The reason for the acceptance of only partially overlapped genes is that evidence based methods might only partially predict a gene, e.g. due to low coverage in RNA-Seq experiments. Hence, requiring a perfect overlap could result in missed predictions. The threshold for overlap acceptance can be set by the user and is also adjustable to only accept 100% overlaps (i.e. perfect overlaps). In the Additional file 1 we show that IPred is robust to different threshold settings and that it is not biased by small overlap thresholds.

If at least two evidence based prediction outputs are available, the former described merging process can be extended by also reporting genes that are not predicted *ab initio*, but have support from different evidence based gene finders. This way, potential novel genes can be identified with greater confidence and also with respect to different approaches and sources for including external information (e.g. RNA-Seq evidence versus EST evidence).

IPred distinguishes between combinations of prokaryotic predictions and eukaryotic predictions since the structure of gene loci can differ substantially depending on the organism type. In contrast to prokaryotes, eukaryotes show splicing events and also alternative splicing resulting in alternative transcript isoforms. This needs to be respected when merging eukaryotic gene predictions. Hence, for each gene locus all corresponding transcripts are processed separately. It is not only important that an exon of a predicted transcript is supported by other methods, but also that the *exon chain* - all neighboring exons - is similar for compared transcripts (because differences indicate an alternative isoform).

Hence, in case of eukaryotes IPred only regards support for an exon of a transcript if the overlapping exon is part of a similar exon chain from a second prediction method. A transcript is classified as “perfect" only if all exons are matched perfectly by a different method. In case all exons of a transcript are supported, but with minor differences (specified by the overlap threshold), the transcript is still classified as “supported" but receives a lower score to indicate less agreement.

If only a part of the exons of a transcript is matched, IPred tests if the overlapping transcripts predicted by other methods have stronger support (i.e. they differ from the currently leading transcript, but agree with each other). If this is the case, the leading transcript is regarded as incorrect and instead the overlapping transcripts with stronger support are taken into account.

If the possibly overlapping transcripts also disagree, the leading transcript is accepted only if the chosen overlap threshold is met by the number of matched exons (for the leading transcript as well as the chosen overlapping other prediction). Since the original overlap threshold is defined as a percentage of nucleotides that need to be covered, the definition of the transcript overlap threshold *t* is slightly adapted: The number of overlapped exons *k* must exceed *t* percent of the total number of exons *n* that are part of the current transcript: *k*≥⌊*t*·*n*⌋.

IPred outputs a new prediction file in GTF format that includes all genes supported by both prediction strategies, categorized by the reliability of each prediction. In addition, a tracking file reports the original genes that generated each merged IPred prediction. Further, additional files reporting genes that were only supported by one strategy are provided, e.g. to allow the analysis of potential novel or not expressed genes. In case a reference annotation is available, all predictions can be automatically evaluated using the framework provided by Cuffcompare [19] to allow an easy comparison of different combinations of gene finders.

It is important to keep in mind that IPred is not intended as a new gene finder but rather as an easy-to-use post processing analysis to verify predictions and to filter out false positives. Therefore, it strongly depends on the quality and performance of the input gene finders, but is independent of the underlying data sets or the nature of the information used for evidence based prediction. Hence, IPred is in principle capable of detecting rare or hard-to-predict events, for instance also ncRNA genes or genes following a non-standard coding scheme, as long as at least some of the input gene finders predict those events.

Currently, IPred returns predictions following the GTF format as interpreted in the Cufflinks suite, e.g. it does not specify UTR features. The reason is that currently the output formats of gene finders strongly differ between methods, and often no UTRs or CDS are reported. Hence, to ensure a broad applicability we decided to be independent of these features and concentrated on gene loci and their corresponding transcripts and used the exon feature to specify coding regions.

Details on the merging process and system requirements are shown in the Additional file 1.

## Experimental setup

We evaluated IPred in four experiments on *E.Coli* (NCBI-Accession: NC_000913.2) and human data (GRCh37). To compare the methods on well-defined known ground truth data, we not only used real data sets but also simulations in our evaluation. In the first *E.Coli* experiment we combined predictions of the widely used *ab initio* gene finders GeneMark [20] and GLIMMER3 [2] and the evidence based gene finders GIIRA [6] and Cufflinks [21]. We simulated RNA-Seq reads based on the NCBI reference annotation of *E.Coli* as evidence information. In the second experiment we used real *E.Coli* RNA-Seq reads (SRA-Accession: SRR546811) as evidence.

The eukaryotic experiments were also analyzed with Cufflinks and GIIRA, as well as with AUGUSTUS [9], a hybrid gene finder that is capable of integrating evidence into its *ab initio* predictions. In the eukaryotic simulation we again used simulated RNA-Seq reads as additional evidence. Further, real RNA-Seq reads (SRA-Accession: SRR1654792) served as evidence for the human real data experiment.

All gene prediction methods were applied in their standard settings, using pre-trained models if available. The incorporation of hints to AUGUSTUS followed the standard pipeline recommended on the AUGUSTUS web page. Note that the use of pre-trained models might introduce a bias favoring the accuracy of *ab initio* based gene finders, due to possible similarities between training data and the data used in this study. However, the comparison of prediction combination methods is unaffected since all combinations are based on the same single method predictions. Full details on the method configurations can be found in the Additional file 1.

The simulation setup uses the read simulator Mason [22] applied to the NCBI reference annotation for each organism of interest. In this annotation the coding sequence of each known isoform appears as a consecutive sequence. Hence, the simulated reads show similar characteristics as real RNA-Seq reads since they cover alternative isoforms, span introns (if existing in the data set) and show a coverage profile typical for gene expression. Note that only 70% of the annotated genes were used for evidence generation, simulating the fact that not all genes are expressed at the same time. The genes that received RNA-Seq support were chosen as the ground truth annotation for the simulation experiments. To obtain a ground truth in the real data experiments, we performed an alignment of the RNA-Seq reads against the reference annotation and counted the number of reads mapping to each annotated gene. Then we regarded all genes as present that received an overall coverage of at least one.

The simulated and real reads were aligned to the reference genome using TopHat2 [23], and the resulting alignment served as evidence for hybrid and evidence based gene prediction. The *ab initio* gene finders were applied directly on the reference genomes.

IPred was used to analyze and combine the resulting gene predictions. We ran Cuffcompare [19] to evaluate all single method predictions and combinations against the ground truth reference annotations. In addition, we compared IPred to gene prediction combinations obtained by Cuffmerge [19] and EVidenceModeler [13], a current state-of-the-art prediction combiner that is an extension of the Combiner idea [15] and was shown to have superior performance to other existing combiners such as GLEAN [14] and JIGSAW [10]. The simulation framework and evaluation is detailed in the Additional file 1.

## Results and discussion

### *E.Coli* simulation

**Figure 1:**
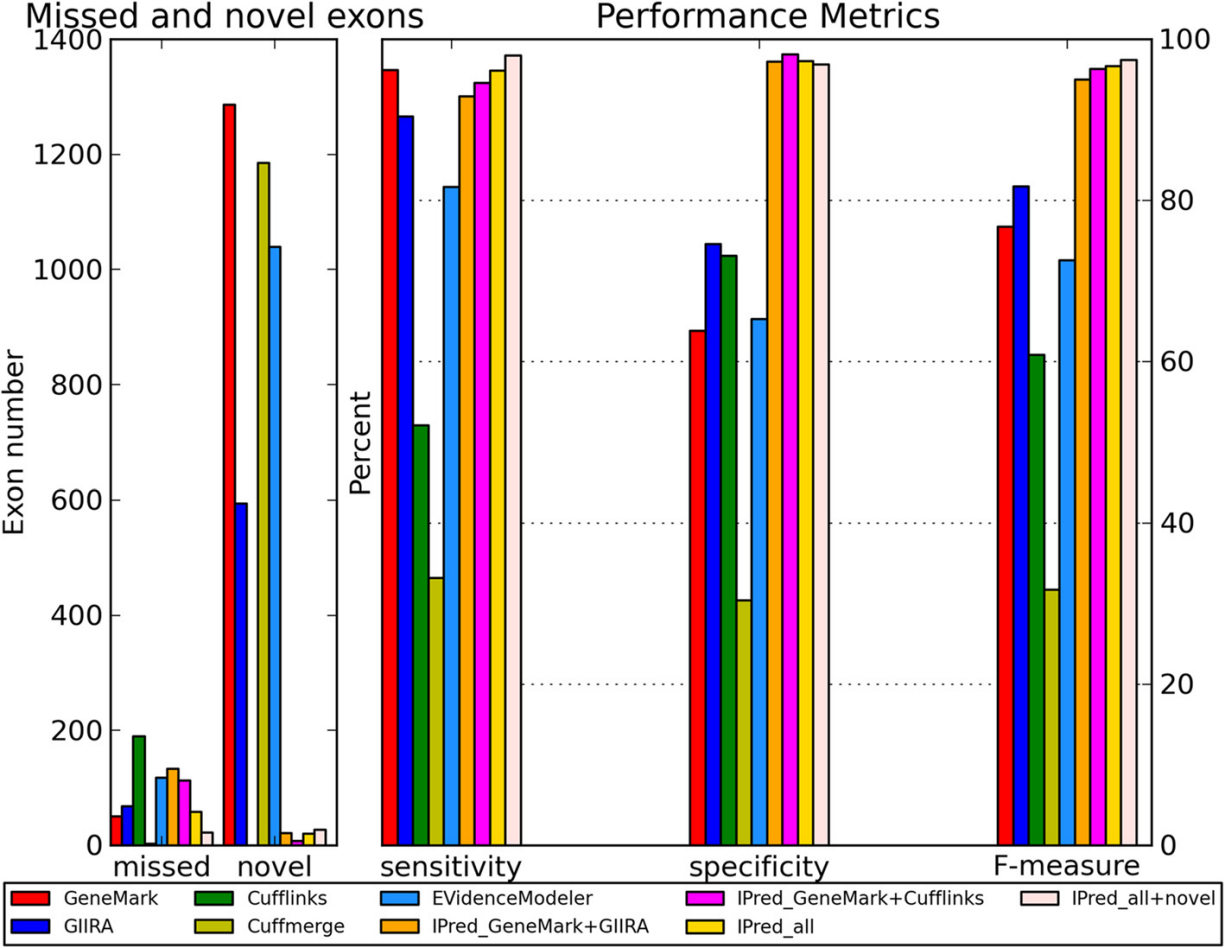
***E.Coli*** **simulation.** Overview of Cuffcompare metrics for the predictions of single methods, EVidenceModeler and IPred combinations for the *E.Coli* simulation. Note that “IPred_all+novel” reports overall supported genes as well as genes missed by GeneMark but supported by both of the evidence based methods.

Figure 1 shows sensitivity, specificity and F-measure (representing the overall prediction accuracy) for the single method gene predictions and different combinations generated by IPred. Overall, we see a large improvement in specificity (e.g. from 63.8% to 98.1% for GeneMark only and GeneMark combined with Cufflinks), while also improving or yielding comparable sensitivity.

Hence, the overall prediction accuracy is significantly improved for combined predictions, as reflected in the considerable higher F-measures. Also the number of missed and novel (not annotated in ground truth, hence false positive) genes is reduced when combining methods. GeneMark and GIIRA originally resulted in a high number of non-annotated predictions. However, when integrating both methods, the merged result shows a substantially reduced number. This indicates that erroneous predictions are filtered out during the merging process because an erroneous prediction by one of the methods is almost always not present in the other method.

Further, we see different effects on prediction accuracy depending on the evidence based method combined with GeneMark predictions. For example, Cufflinks appears to yield a set of predictions that is more complementary to GeneMark than GIIRA predictions because the combination of GeneMark and Cufflinks leads to a higher sensitivity and fewer missed exons than GeneMark combined with GIIRA.

The significant accuracy improvement of combinations with Cufflinks in comparison to predictions from Cufflinks alone is due to the fact that Cufflinks does not predict structural genes but only the expressed transcript. Hence, its original sensitivity and specificity are comparably low, but are substantially increased when combined with other methods predicting structural genes.

Although the combination of two gene finders already results in improved accuracy, the combination of all three methods yields the most accurate results. Further, when also genes missed by GeneMark but supported by both of the evidence based methods are taken into account, we note an additional increase in sensitivity while showing comparable specificity.

Independently of the chosen combination IPred outperforms EVidenceModeler and Cuffmerge with significantly increased sensitivity and specificity. Cuffmerge and in some cases also EVidenceModeler even results in smaller sensitivity and specificity compared to the single method predictions. We assume that the low accuracy of Cuffmerge is due to the fact that it does not include any weighting of different prediction methods, as opposed to EVidenceModeler and the strategy combination of IPred. A more detailed analysis of the *E.Coli* evaluation is shown in the Additional file 1.

### Human simulation

**Figure 2:**
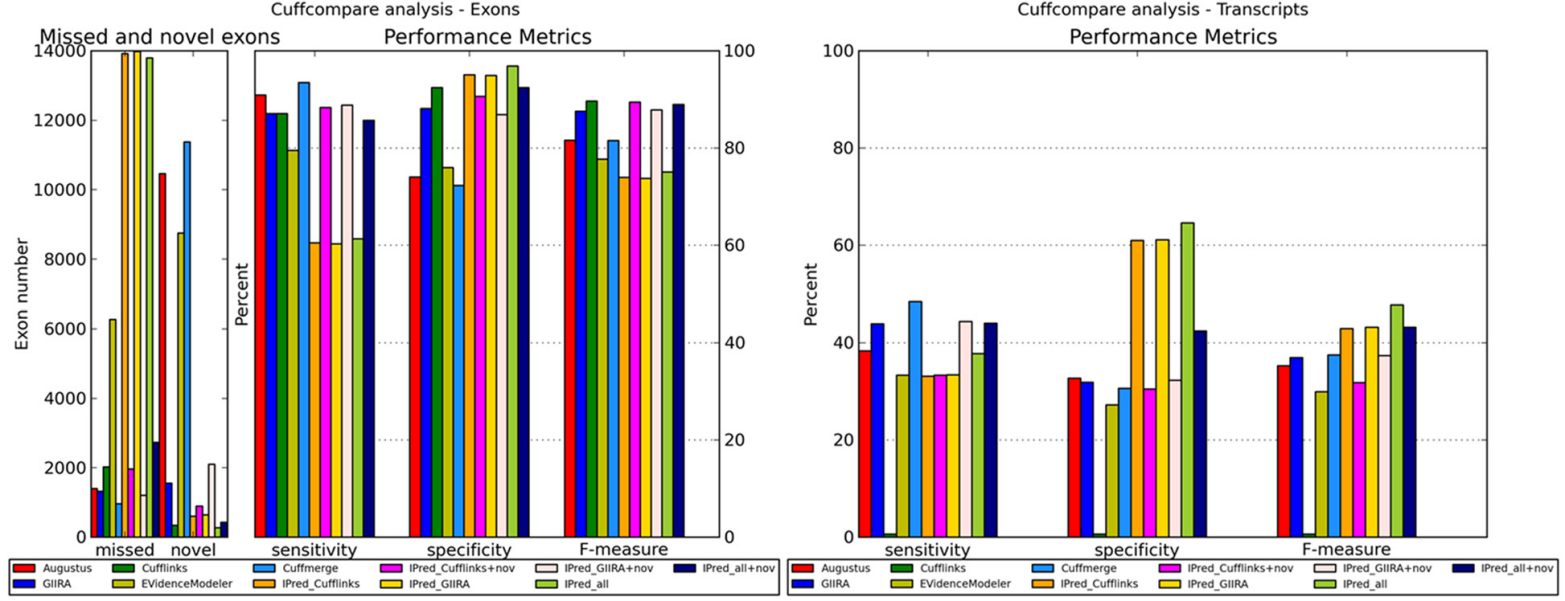
**Human simulation.** Overview of Cuffcompare metrics for the predictions of single methods and method combinations for the simulated human data set. On the left-hand side the exon level evaluation is shown, on the right-hand side the comparison on the transcript level. Note that “IPred_all+nov" reports overall supported genes as well as genes missed by AUGUSTUS but supported by both of the evidence based methods.

IPred was also tested on a simulation of a eukaryotic dataset based on chromosomes 1, 2 and 3 of the human genome. Figure 2 shows the exon and transcript level comparison of the single method prediction accuracy with IPred, EVidenceModeler and Cuffmerge combinations. Details are shown in the Additional file 1.

Overall, we see that the performance on exon and transcript level significantly differs between methods. On the exon level, the sensitivity of IPred combinations strongly depends on the integration of novel predictions. If only predictions present in both AUGUSTUS and one or two of the evidence based methods are taken into account, the sensitivity is significantly reduced compared to all other combinations. At the same time the specificity is on a comparable or slightly higher level compared to other IPred combinations, and significantly higher than the EVidenceModeler and Cuffmerge prediction. This results in an accuracy comparable to EVidenceModeler but substantially decreased in comparison to other IPred combinations and also decreased in comparison to the single method predictions.

If predictions are included that do not overlap with AUGUSTUS identifications (either not sufficiently with respect to the overlap threshold or not at all), the sensitivity significantly increases, together with only slight decrease in specificity. Hence, these IPred combinations clearly outperform the result of EVidenceModeler. Also the Cuffmerge combinations are outperformed since the high sensitivity of Cuffmerge is accompanied with significantly lower specificity.

The reason for the performance difference between combinations including and excluding “novel” predictions is that AUGUSTUS often reports a transcript with an incorrect first or last exon (i.e. it reports an additional exon, data not shown). This is also reflected in the high number of novel exons predicted by AUGUSTUS and in its low specificity. Though a detailed analysis of this phenomenon is beyond the scope of this work, a likely explanation is that AUGUSTUS is in principle an *ab initio* gene finder and only integrates hints from other evidence. The additional exons might be an artifact due to the *ab initio* prediction (that also predicts genes that are not expressed in our simulation). Hence, in combinations with Cufflinks and GIIRA the exon chains of the compared methods disagree and none of the predictions appears to be sufficiently supported.

Although including novel genes significantly increases the sensitivity, IPred is still affected by the discrepancies between AUGUSTUS and evidence based predictions because it shows a sensitivity only comparable to Cufflinks and GIIRA and therefore yields comparable results in the overall accuracy.

All in all, on the exon level IPred (including genes not fully supported by AUGUSTUS) yields more accurate results than EVidenceModeler and Cuffmerge and comparable results to the best single methods.

On the transcript level Cufflinks as the best performing method on the exon level yields almost no perfectly predicted transcripts. This is due to the fact that Cufflinks is very accurate at predicting intermediate exons but does not predict start and stop codons and therefore beginning and the end of a transcript almost never match the reference annotation. Here, IPred proves very useful because it can complement the overall exon accuracy of Cufflinks with the start and stop prediction accuracy of other methods, which is reflected in the strong increase in sensitivity and specificity compared to Cufflinks alone.

Hence, IPred again yields more accurate predictions than EVidenceModeler and Cuffmerge. Further, it also increases the accuracy of the single method predictions. Detailed values of the Cuffcompare evaluation are shown in the Additional file 1.

In addition, we compared the performance of the three gene prediction combination methods in regard to memory consumption and speed. Table 1 shows the peak memory and overall time necessary to analyze and combine the single method predictions. IPred has the lowest memory and running time requirements of the three compared gene prediction combination methods.

**Table 1:** **Overall running time (in seconds) and peak memory (in megabytes) for the compared gene prediction combination methods to analyze the simulated human data set**

**Performance evaluation**		
**Combination method**	**Overall time (s)**	**Peak memory (MB)**
EVidenceModeler	23 037	3 100
Cuffmerge	132	624
IPred	59	215

### *E.Coli* real data set

**Figure 3:**
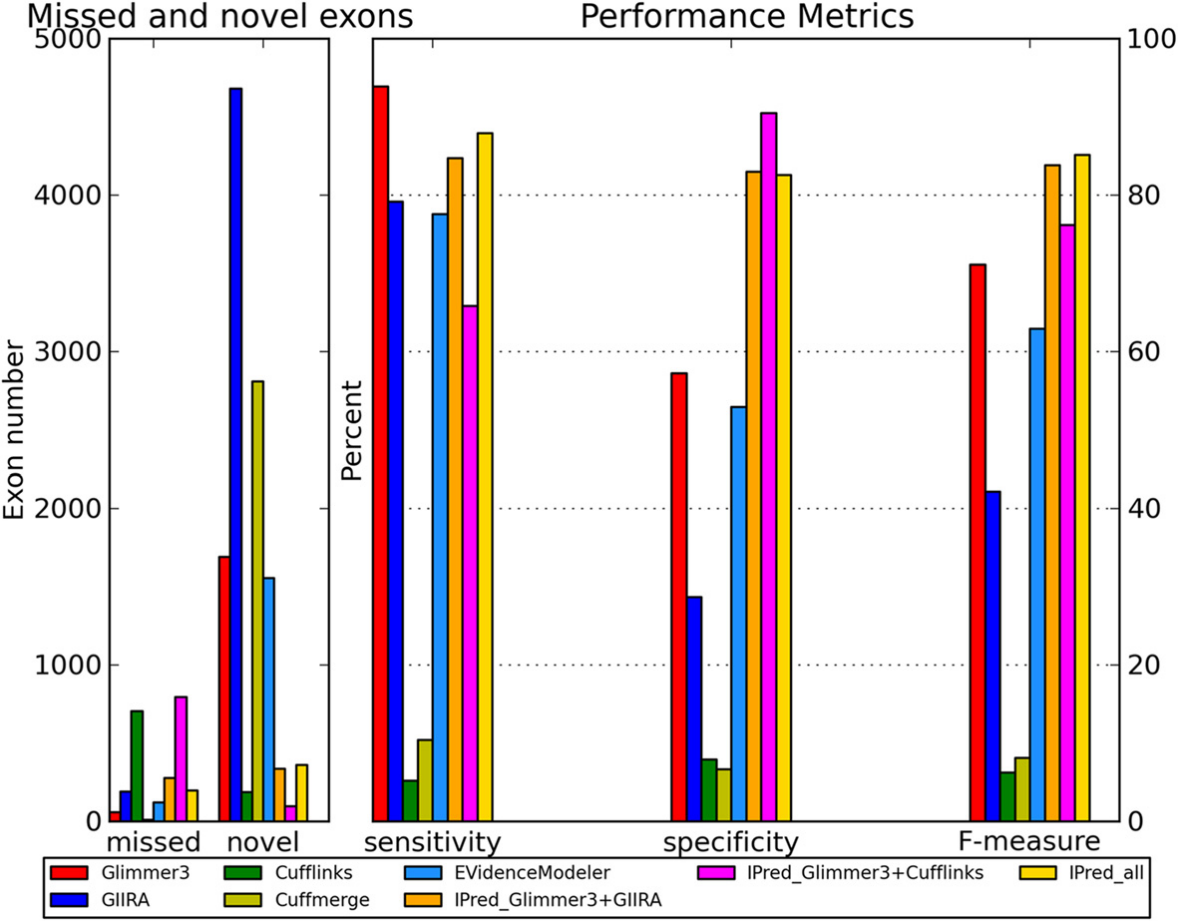
***E.Coli*** **real data.** Overview of Cuffcompare metrics for the predictions of the single methods, EVidenceModeler, Cuffmerge, and IPred combinations for the real *E.Coli* data set.

For further evaluation we also applied IPred to *E.Coli* predictions based on real RNA-Seq evidence. Figure 3 shows the results of the Cuffcompare evaluation against the subset of likely expressed reference annotations. A more detailed analysis can be found in the Additional file 1.

Overall, IPred combinations show a pronounced increase in specificity and result in significantly improved prediction accuracy compared to all other methods. Glimmer3 shows the highest sensitivity of all methods. Since none of the approaches that include RNA-Seq evidence show a comparable sensitivity, this is likely due to the choice of the ground truth annotation set that might still contain genes that are not expressed but are rather mapping artifacts. Here, including other evidence, such as protein alignments, might even further increase the accuracy of combined predictions. Cufflinks and also Cuffmerge show very low accuracy, which indicates that they are more suitable for application on eukaryotes than on prokaryotes.

### Human real data set

**Figure 4:**
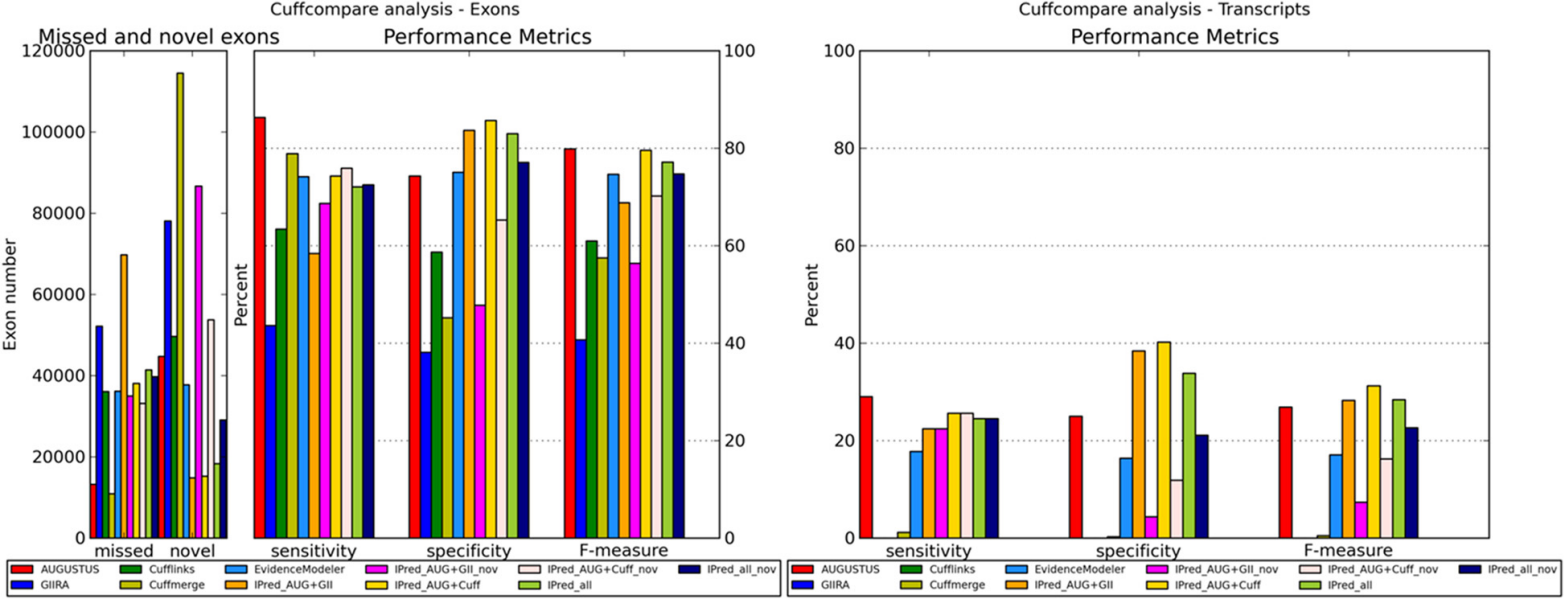
**Human real data.** Overview of Cuffcompare metrics for the predictions of single methods and method combinations for the complete human real data set. On the left-hand side the exon level evaluation is shown, on the right-hand side the comparison on the transcript level. Note that “IPred_all+nov" reports overall supported genes as well as genes missed by AUGUSTUS but supported by both of the evidence based methods.

The results of the evaluation on a complete human data set with real RNA-Seq reads are shown in Figure 4 and are detailed in the Additional file 1.

On exon as well as transcript level AUGUSTUS shows the highest prediction sensitivity, while the IPred combinations (without including potential novel genes) show the highest specificity. This further demonstrates that IPred successfully excludes false positive predictions.

However, on the exon level the overall accuracy of AUGUSTUS predictions (79.9%) is slightly higher than the accuracy of combinations by IPred based on Cufflinks or Cufflinks and GIIRA (79.6% and 77.2%).

On the transcript level the difference in sensitivity is not as pronounced as on the exon level. Hence, here the overall accuracy of IPred predictions (without potential novel genes) is slightly higher than for AUGUSTUS, due to the improved specificity of IPred.

On this data set including potentially novel genes also resulted in higher sensitivity (on the exon level more pronounced than on the transcript level); however, at the cost of reduced specificity and overall reduced accuracy. This indicates that although Cufflinks and GIIRA agree on certain expressed regions, these predictions still require further analysis to ensure that they are not mapping artifacts. However, these regions might also hint to novel genes, but additional evidence, for instance from ESTs or protein libraries would be necessary for further verification, which is beyond the scope of this manuscript.

In comparison with Cuffmerge and EVidenceModeler, IPred shows improved prediction accuracy, in particular on the transcript level. On the exon level, combinations by EVidenceModeler are comparable to IPred. Cuffmerge shows the highest exon level sensitivity of all combination methods, but at the cost of the lowest specificity.

## Conclusion

Gene prediction is an important task in genome analysis pipelines. Existing approaches aim at accurately identifying genes based on varying strategies, categorized as *ab initio* and evidence based. Since both types have their own strengths and weaknesses, combining *ab initio* and evidence based methods can complement advantages to improve the overall prediction accuracy. We developed IPred, an automated pipeline to integrate the results of gene prediction methods based on different strategies to enhance the accuracy of the combined gene identifications. The benefit is shown in simulations and on real data: IPred shows favorable sensitivity and specificity compared to single method predictions and to existing combiners.

Thus, we believe that IPred is a valuable addition to the set of existing methods for prediction combination. It is an easy-to-use software and allows broad flexibility on combined prediction results.

## Availability and requirements

**Project name:** IPred.**Project home page:**https://sourceforge.net/projects/ipred/.**Operating systems:** Platform independent.**Programming language:** Python (main software) and Java (GUI).**Other requirements:** Java 7 is required for using the GUI. Further, for using IPred from source, Python 2.7 or higher and the packages Matplotlib and Numpy are required.**License:** GNU Lesser General Public License, version 3.0.**Any restrictions to use by non-academics:** No.

## Additional file

Additional file 1 **Supplementary material to IPred.** PDF document containing supplementary information on methods, system requirements, experimental setup, and results.
