# Supplementary material for: IPred - integrating ab initio and evidence based gene predictions to improve prediction accuracy
Source: BMC Genomics. 2015 Feb 26;16(1):134. doi: 10.1186/s12864-015-1315-9 (PMC4345001; doi:10.1186/s12864-015-1315-9)
Supplement: Supplementary file 1 — Supplementary material to IPred. PDF document containing supplementary information on methods, system requirements, experimental setup, and results. [file 12864_2015_1315_MOESM1_ESM.pdf]

Supplementary Material for IPred - Integrating Ab Initio and Evidence Based Gene Predictions to Improve Prediction Accuracy

Franziska Zickmann and Bernhard Y. Renard
Research Group Bioinformatics (NG4) Robert Koch-Institute, 13353 Berlin, Germany

1 System Requirements

IPred is written in python (version 2.7, http://www.python.org/ with additional packages Matplotlib and Numpy) and was designed and tested on a linux system. Precompiled executables are available for linux, windows and mac. The GUI (refer to Figure 1 for a screenshot) is implemented in Java 7 (http://www.java.com) and is available as a precompiled jar file that is executable on linux, windows and mac.

IPred required approximately 15s and 50MB of memory to analyze the combinations of four different gene prediction outputs for the *E.Coli* genome and approximately 59s and 215MB of memory to analyze the combinations of three gene prediction methods for the eukaryotic simulation on human chromosomes 1, 2, and 3.

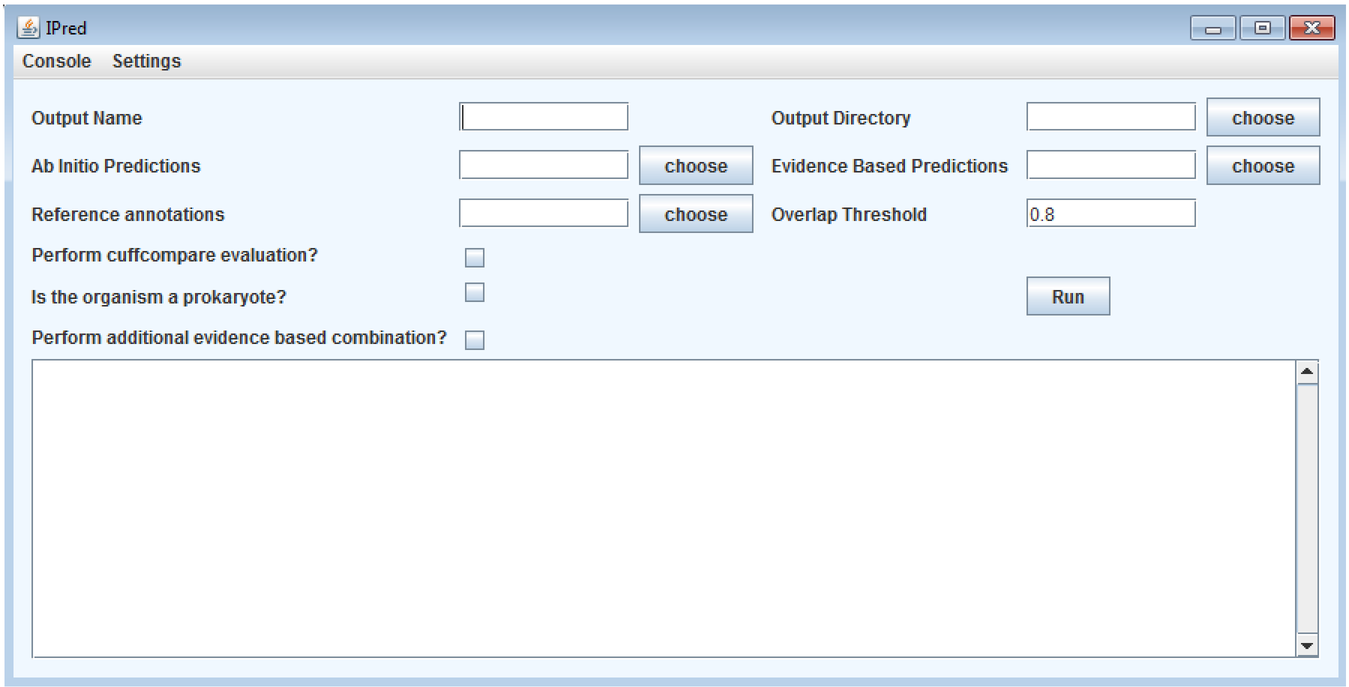

Figure 1: Screenshot of the IPred GUI.

2 Methods

In the following, we present a more detailed view on the process of merging the output of two or more prediction methods to complete Section 2 of the manuscript.

IPred categorizes the reported gene predictions depending on the quality of the overlap with other predictions. If start and stop position of both *ab initio* and evidence based predictions are equal, this gene is regarded as strongly supported and is ranked in the best category ("perfect", prediction score=1.0). If the gene is sufficiently overlapped (depending on the defined overlap threshold) but not perfectly matched, it receives a lower score that is calculated as the length of overlapped bases divided by the total length of the gene.
The last category comprises genes that are only predicted by one of the compared methods, i.e. "novel" genes. These predictions are written to additional output files corresponding to their prediction strategy and receive the lowest score of 0.0.

When supported predictions are written to the new GTF file, the *ab initio* identifications are regarded as the leading predictions, which means that they define start and stop position of the gene. However, IPred also has the option to regard the evidence based prediction as the leading one (option: -b).

3 Experimental Setup

We tested IPred in a simulation based on the *E.Coli* genome (NCBI-Accession: NC_000913.2) and combined predictions of the *ab initio* gene finders GeneMark (Besemer *et al.*, 2001) (GeneMark.hmm PROKARYOTIC, Version 2.10f) and Glimmer3 (Delcher *et al.*, 2007) and the evidence based gene finders GIIRA (Zickmann *et al.*, 2014) and Cufflinks (Trapnell *et al.*, 2010) (version 2.0.2). GeneMark and Glimmer3 have been applied directly on the genomic sequence, without the need for integration of simulation data. For evidence based predictions we simulated RNA-Seq reads based on the NCBI reference annotation, as explained in the following subsections.
Further, we applied the gene prediction combiner EVidenceModeler (Haas *et al.*, 2008) (version of 25th June 2012) and Cuffmerge (Trapnell *et al.*, 2012) (version 1.0.0) on the obtained predictions to compare the accuracy of IPred combinations with existing combiners.

In the second *E.Coli* experiment we used real RNA-Seq reads (SRA-Accession: SRR546811) as evidence for GIIRA and Cufflinks. Again we compared IPred with gene prediction combinations from EVidenceModeler and Cuffmerge. Since for this experiment no predefined ground truth is available, we used a subset of annotated genes that had support of reads and can therefore be regarded as expressed (refer to the following subsections for details).

The eukaryotic simulation is based on chromosomes 1,2 and 3 of the human genome assembly GRCh37 (NCBI-Accessions: NC_000001.10, NC_000002.11, NC_000003.11). Here we applied the hybrid eukaryotic gene prediction method AUGUSTUS (Stanke *et al.*, 2006) and the evidence based methods GIIRA and Cufflinks. Similarly to the prokaryotic

simulation, we used evidence in form of simulated RNA-Seq reads (details are below). Further, we applied EVidenceModeler and Cuffmerge to obtain combined predictions of the three prediction methods.
In addition, we tested the six methods on a human real data set (SRA-Accession of RNA-Seq reads: SRR1654792). As for the prokaryotic real data set, we sampled a subset of annotated genes as ground truth reference (details below).
Although we applied various single gene prediction tools, for the ease of visualization and comparison we focused on the best suited single gene prediction method for each data set (AUGUSTUS for human, Glimmer3/GeneMark for *E.Coli*).

GeneMark We used the script gmsn.pl provided in the GeneMark installation to perform the *ab initio* gene prediction:

>perl gmsn.pl -name gmS_ecoli -prok EColi_MG1655_NC0009132.fasta

Afterwards, the resulting .fasta.lst file was converted to GTF format using the script convertGeneMark.py provided with the IPred installation:

> python convertGeneMark.py ecoliGeneMark.fasta.lst ecoliGeneMark.gtf

Glimmer3 We used the script g3-from-scratch.csh provided in the Glimmer3 installation that automatically finds a set of training genes and then runs Glimmer3 on the result:

>./g3-from-scratch.csh EColi_MG1655_NC0009132.fasta ecoliGlimmer

Afterwards, the resulting .predict file was converted to GTF format using the script convertGlimmer.py provided with the IPred installation:

> python convertGlimmer.py ecoliGlimmer.predict ecoliGlimmer.gtf

Simulation Study As evidence information for the *E.Coli* data set we simulated Illumina RNA-Seq reads with a length of 36bp based on the NCBI reference annotation (http://www.ncbi.nlm.nih.gov/). Note that only 70% of the annotated genes were used for evidence generation, simulating the fact that not all genes are expressed at the same time. Therefore, we randomly picked 2902 out of the present 4146 annotations and used the chosen fasta sequences as input for the next-generation sequencing read simulator Mason (Holtgrewe, 2010).
Before applying Mason, the set of annotated coding sequences was divided into 3 parts with 1451, 1016 and 435 genes, respectively. These three subsets were separately simulated with different coverages (20, 5 and 25, respectively), to obtain different gene expression levels in the afterwards combined set of simulated RNA-Seq reads.

Similar to the prokaryotic simulation, we simulated Illumina RNA-Seq reads with a length of 50bp based on the NCBI reference annotation for GRCh37 chromosomes 1, 2 and 3. Also here we only simulated approximately 70% of the genes as expressed and generated varying coverage levels ranging from nucleotide coverage 5 to 20. This resulted in 5318

genes that have RNA-Seq evidence (2482, 1488 and 1348 for chromosome 1, 2 and 3, respectively), out of 7596 annotated reference genes (3545, 2126 and 1925 for chromosome 1, 2 and 3, respectively).

Originally we intended to use the same read length in our simulated experiments, but due to few very short exons in the *E.Coli* data set it was not possible to simulate 50bp reads for *E.Coli* (because then the read length would have exceeded the length of the gene). However, 50bp is a better reflection of current RNA-Seq read lengths than 36bp, so we decided to not reduce the length of reads in the human simulation.

In both the prokaryotic and eukaryotic simulation the sample of genes selected as expressed served as the ground truth annotation. All genes that are predicted and do not match this ground truth are regarded as false positives (or “novel exons” throughout the manuscript and in the Cuffcompare analysis), independent of the fact that the predicted gene locus might be present in the remaining (unexpressed) NCBI reference genes.

Ground truth for the real data sets Since not all genes of *E.Coli* and *Homo sapiens* are necessarily expressed at the same time, we performed the evaluation by comparing against a subset of likely expressed reference genes. We note that this subset does not necessarily reflect an exact ground truth but is only intended as an approximation of the real ground truth that serves as a basis to evaluate the performance of the compared methods.
To obtain the subset, we mapped the RNA-Seq reads against the NCBI reference transcripts, using Bowtie2 (Langmead and Salzberg, 2012) (version 2.2.1) with default parameters (it was not necessary to use TopHat2, because no split read mapping is required).

E.Coli
```
> bowtie2-build EColi_cds.fasta EColi_cds
> bowtie2 -S mapping_ecoliReal.sam –no-unal -q -x EColi_cds -U SRR546811.fastq
```
Human
```
> bowtie2-build HG19_cds.fasta HG19_cds
> bowtie2 -S mapping_HumanReal.sam –no-unal -q -x HG19_cds -U SRR1654792.fastq
```

We analyzed all resulting alignments and counted all reads mapping to each annotated gene. Then we sampled a subset of reference genes comprising all annotations with a minimum overall mapping coverage of one (the average mapping coverage was 17). For *E.Coli* this resulted in a ground truth sample of 2680 reference genes instead of the original 4146 annotations. For the human data set this resulted in 19124 instead of 34074 genes.

Read mapping We applied the read mapper TopHat2 (Kim *et al.*, 2013) (version 2.0.8) to obtain a mapping of the RNA-Seq reads on the *E.Coli* genome and the human chromosomes, respectively. We first indexed the reference sequence with Bowtie2 and then called TopHat2 with default settings on the reference and the corresponding RNA-Seq reads in fastq format:

```
> bowtie2-build EColi_MG1655_NC0009132.fasta EColi_MG1655_NC0009132
> tophat2 EColi_MG1655_NC0009132 ecoliReads.fastq
```

```
> bowtie2-build chr123.fasta chr123
> tophat2 chr123 humanReads.fastq
```

```
> bowtie2-build hg19_chromosomes.fasta hg19_chromosomes
> tophat2 hg19_chromosomes humanReads.fastq
```

The details of the mappings are shown in Table 1.
The resulting mappings were then analyzed by GIIRA and Cufflinks to obtain the evidence based gene predictions. Further, the mapping for the human simulation was used to generate hints for AUGUSTUS gene predictions.

data set	reads mapped	ambiguous reads	hits total	average mapping coverage
E.Coli simulation	1,187,830	16,019	1,253,150	16.6
E.Coli real	10,052,045	8,555,561	57,769,265	17
human simulation	3,122,322	140,749	3,497,908	22.14
human real	126,914,607	9,340,757	142,753,401	293

Table 1: Table showing the general properties of the TopHat2 mapping of the simulated and real data reads to the *E.Coli* genome and to the human data sets.

Cufflinks In all four experiments Cufflinks was applied directly on the resulting mapping file (in BAM format), using default settings:

```
> cufflinks mappingTopHat.bam
```

GIIRA For the analysis with GIIRA, in all four experiments first we used samtools (Li *et al.*, 2009) to sort the BAM file according to read names and then to convert the BAM file into SAM format:

```
> samtools sort -n mappingTopHat.bam mappingTopHat_sorted
> samtools view -h -o mappingTopHat_sorted.sam mappingTopHat_sorted.bam
```

Finally, GIIRA was applied with default settings, using CPLEX (CPLEX, 2011) as the optimization method:

Prokaryotic data sets:

```
> java -jar GIIRA.jar -iG EColi_MG1655_NC0009132.fasta -haveSam
mappingTopHat_sorted.sam -prokaryote
```

Eukaryotic simulation:
> java -jar GIIRA.jar -iG chr123.fasta -haveSam mappingTopHat_sorted.sam

Human real data set, using a minimal coverage of 1:
> java -jar GIIRA.jar -iG hg19_chromosomes.fasta -minCov 1
-haveSam mappingTopHat_sorted.sam

Further, the GIIRA output was post-processed using the filter script “filterGenes.py” provided and recommended in the GIIRA installation.

> python filterGenes.py GIIRAgenes.gtf GIIRAgenes_filtered.gtf y y y

AUGUSTUS AUGUSTUS is able to incorporate information from RNA-Seq experiments in the form of “external hints”. To integrate the evidence we followed the pipeline recommended on the AUGUSTUS website[1]. We filtered the TopHat2 mappings to only contain uniquely mapped reads and generated hints from the remaining alignments, which were then included in the AUGUSTUS prediction:

Simulation:
> augustus –species=human –extrinsicCfgFile=extrinsic.M.RM.E.W.cfg
–alternatives-from-evidence=true –hintsfile=hintsAUG.gff
–allow_hinted_splicesites=atac chr123.fasta > augHuman123.out

Real data set:
> augustus –species=human –extrinsicCfgFile=extrinsic.M.RM.E.W.cfg
–alternatives-from-evidence=true –hintsfile=hintsAUG.gff
–allow_hinted_splicesites=atac hg19_chromosomes.fasta > augHumanReal.out

IPred IPred analyzed and combined the input predictions with the parameter settings “-p” to indicate a prokaryotic dataset (only in case of the *E.Coli* experiments) and “-f” to perform the Cuffcompare evaluation:

Prokaryotic data sets:
> python IPred.py -a ecoli_ref_cds_70percent.gtf -i input_abInitio.txt
-e input_evBased.txt -p -f

Eukaryotic simulation:
> python IPred.py -a chr123_70percent.gtf -i input_abInitio.txt -e input_evBased.txt -f
Human real data set:

[1]http://bioinf.uni-greifswald.de/bioinf/wiki/pmwiki.php?n=Incorporating
RNAseq.Tophat

> python IPred.py -a hg19_cov1.gtf -i input_abInitio.txt -e input_evBased.txt -f -t 0.3
The overlap threshold of 0.3 allows to balance variances of start and stop predictions between methods.

The files "input_abInitio.txt" and "input_evBased.txt" contain the name and location of the prediction methods that shall be combined by IPred.

EVidenceModeler As required of EVidenceModeler we created an evidence weights file to indicate the input predictions and their associated weights. The type of GeneMark and of AUGUSTUS was specified as "ABINITIO_PREDICTION" and Cufflinks and GIIRA predictions were declared as "OTHER_PREDICTION" (because EVidenceModeler provided no explicit type for evidence based predictions but instead recommends to use "OTHER_PREDICTION" for complete gene predictions other than *ab initio*).
In the prokaryotic simulation the predictions of GeneMark, GIIRA and Cufflinks and also Glimmer3, GIIRA and Cufflinks were combined, while in the eukaryotic simulation and real data set AUGUSTUS was combined with GIIRA and Cufflinks. For the real *E.Coli* data set, Glimmer3 was combined with GIIRA and Cufflinks, where all prediction have equal weights.
For each simulation experiment we performed two runs with EVidenceModeler, one with equal weights (= 1) for each of the methods, and one with higher weights for the evidence based predictions (= 5 for the prokaryotic simulation, = 3 for the eukaryotic simulation because AUGUSTUS also received RNA-Seq hints) to indicate their presumably higher reliability due to the use of RNA-Seq information. Refer to Section 4.5 for an analysis of the influence of the choice of weights.
Each run was performed following the pipeline recommended on the EVidenceModeler webpage (http://evidencemodeler.sourceforge.net/).

Cuffmerge In all four experiments Cuffmerge was applied on an input file specifying the paths to the respective gene predictions. In the prokaryotic simulation the predictions of GeneMark, GIIRA and Cufflinks and also Glimmer3, GIIRA and Cufflinks were combined, while in the eukaryotic simulation and real data set AUGUSTUS was combined with GIIRA and Cufflinks. For the real *E.Coli* data set, Glimmer3 was combined with GIIRA and Cufflinks.

> cuffmerge -o cuffmerge_output inputCuffmerge.txt

Evaluation framework IPred analyzed and combined the resulting gene predictions and used Cuffcompare (Trapnell *et al.*, 2012) to evaluate the single method predictions and the combinations against the ground truth reference annotations. Cuffcompare is based on the guidelines presented in (Burset and Guigó, 1996) and evaluates the sensitivity and specificity of a set of gene predictions in regard to several levels, namely the base, exon, intron, intron-chain, transcript, and locus level. For our prokaryotic data sets we focused on the comparison of the exon level since a gene in the NCBI reference annotation corresponds to an exon in the Cuffcompare evaluation (and hence exon and transcript level

are equal). For the eukaryotic prediction we compared the exon as well as the transcript level.
Each prediction can be a correct prediction as part of a coding sequence ("True positive" or TP) or as non-coding ("True negative" or TN) or vice versa a false prediction as coding ("False positive" or FP) or non-coding ("False negative" or FN).
Now, sensitivity (Sn) and specificity (Sp) can be obtained by calculating the proportion of true predictions on the set of all possible coding reference genes and the set of all predicted genes, respectively:

$$Sn = \frac{TP}{TP + FN} \tag{1}$$

$$Sp = \frac{TP}{TP + FP}. \tag{2}$$

Cuffcompare also reports the number of reference exons that have been missed by the prediction method ("missed exons") and the number of exons that have been predicted but do not match any of the reference annotations ("novel exons").

As a widely used representative value of overall prediction accuracy we further calculate the F-measure (van Rijsbergen, 1979) F to provide a combined measure of sensitivity (Sn) and specificity (Sp):

$$F = 2 \cdot \frac{Sp \cdot Sn}{Sp + Sn}. \tag{3}$$

4 Results

4.1 *E.Coli* simulation

To complete the results shown in the manuscript, in the following we present a more detailed analysis of the former presented combination of GeneMark predictions with GIIRA and Cufflinks and also provide a second set of combinations using the *ab initio* gene finder Glimmer3 (Delcher *et al.*, 2007). Refer to Table 2 for a summary of the absolute numbers and percentages of sensitivity and specificity of the Cuffcompare evaluation for both GeneMark and Glimmer3 combinations. Note that for better visibility we included the Cuffmerge results only in Table 2 but not in the accompanying figures, since Cuffmerge led to the least accurate results.

(1) GeneMark combinations

method	missed	novel	sensitivity	specificity	Fmeasure
GeneMark	51	1286	96.2	63.8	76.7
Cufflinks	190	**0**	52.1	73.1	60.8
GIIRA	68	594	90.4	74.6	81.7
IPred_Cufflinks	113	8	94.6	**98.1**	96.3
IPred_Cufflinks+novel	76	8	96.3	98.0	97.1
IPred_GIIRA	133	21	92.9	97.2	95.0
IPred_GIIRA+novel	89	117	95.2	93.0	94.1
IPred_Cufflinks+GIIRA	58	20	96.1	97.3	96.7
IPred_Cufflinks+GIIRA+novel	22	27	**98.0**	96.9	**97.4**
EVidenceModeler	118	1039	81.7	65.3	72.6
Cuffmerge	**3**	1185	33.2	30.4	31.7

(2) Glimmer3 combinations

method	missed	novel	sensitivity	specificity	Fmeasure
Glimmer3	78	1336	94.0	62.0	74.7
Cufflinks	190	**0**	52.1	73.1	60.8
GIIRA	68	594	90.4	74.6	81.7
IPred_Cufflinks	142	12	92.2	**96.5**	94.3
IPred_Cufflinks+novel	72	12	95.3	96.2	95.7
IPred_GIIRA	163	20	90.8	96.0	93.3
IPred_GIIRA+novel	87	119	95.0	91.8	93.4
IPred_Cufflinks+GIIRA	88	20	93.9	96.1	95.0
IPred_Cufflinks+GIIRA+novel	23	35	**97.6**	95.2	**96.4**
EVidenceModeler	127	944	81.4	66.9	73.4
Cuffmerge	**2**	1218	32.6	29.6	31.0

Table 2: These two tables present the absolute numbers and percentages of the Cuffcompare evaluation of the exon level for the combinations based on GeneMark and Glimmer3 (Table 2.(1) and Table 2.(2), respectively). Note that all IPred combinations include either GeneMark (1) or Glimmer3 (2). The best values for each category are marked in bold.

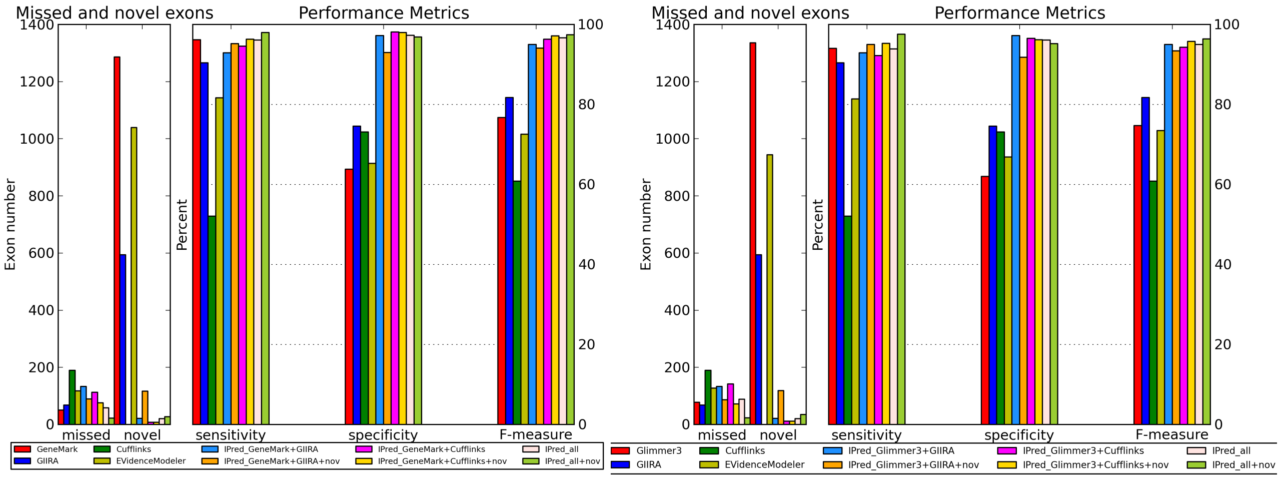

(a) GeneMark-based (b) Glimmer3-based

Figure 2: Overview of Cuffcompare metrics for the predictions of single methods and the method combinations for the *E.Coli* simulation. In (a) the combinations all include GeneMark, in (b) they include Glimmer3. Note that "+nov" indicates that this combination not only reports the genes supported by all combined methods but also novel ones that have been predicted by the used evidence based methods.

Supplementary Figure 2(a) is an extension of Figure 1 of the manuscript since here also two-method combinations are shown that are complemented with novel predictions from only one used evidence based method (i.e. "IPred_GeneMark+Cufflinks+nov" and "IPred_GeneMark+GIIRA+nov"). These novel predictions are identifications that are not supported by the *ab initio* method but are only indicated by the evidence based approaches. We see that including these novel genes (that have no support from any other evidence based prediction) results in an improved sensitivity, though at the cost of decreased specificity. In case of the IPred_GeneMark+Cufflinks+novel combination the loss in specificity is only small. This is due to the fact that Cufflinks yields no completely novel exons, hence erroneous predictions only arise if Cufflinks predicts an exon that does not perfectly match the reference annotation.
We note that only including novel predictions that have support from more than one evidence based method yields more accurate results, i.e. a pronounced improvement in sensitivity while also preserving specificity. This indicates that combining two or more evidence based methods is a suitable method to further verify predictions and to avoid a loss in specificity that accompanies a simple integration of all novel predictions.

Figure 2(b) shows the Cuffcompare evaluations for the combinations with the gene finder Glimmer3. In general, this experiment shows the same trends as the GeneMark combinations, we see an improved prediction accuracy when combining different prediction strategies. We also note that combining three methods yields better results than combining two methods since here we can include genes that are not supported by the *ab initio* method but from two evidence based methods.

Further, the compared method EVidenceModeler is again outperformed by all IPred combinations, which yield a better sensitivity and specificity and hence a higher F-measure. Compared to GeneMark, Glimmer3 shows a slightly reduced prediction accuracy. This is also reflected in combinations with Glimmer3, which have slightly lower F-measures than combinations based on GeneMark.

4.2 Human simulation

To complement the eukaryotic evaluation of the manuscript, Table 3 shows the details on the actual numbers and percentages of the Cuffcompare evaluation.

Human simulation

			Exon			Transcript		
method	missed	novel	Sn	Sp	F	Sn	Sp	F
AUGUSTUS	1401	10458	90.9	74.0	81.6	38.3	32.7	35.3
GIIRA	1320	1551	87.1	88.1	87.6	43.9	31.9	37.0
Cufflinks	2026	344	87.1	92.4	**89.7**	0.6	0.6	0.6
IPred_Cufflinks	13921	600	60.5	95.1	74.0	33.1	61.0	42.9
IPred_Cufflinks+nov	1965	890	88.3	90.6	89.4	33.3	30.5	31.8
IPred_GIIRA	13977	640	60.3	94.9	73.7	33.4	61.1	43.2
IPred_GIIRA+nov	1208	2101	88.8	86.9	87.8	44.4	32.3	37.4
IPred_Cufflinks+GIIRA	13792	**275**	61.3	**96.9**	75.1	37.8	**64.6**	**47.7**
IPred_Cufflinks+GIIRA+nov	2736	430	85.7	92.4	88.9	44.0	42.4	43.2
EVidenceModeler	6274	8753	79.5	76.0	77.7	33.3	27.2	29.9
Cuffmerge	**965**	11375	**93.5**	72.3	81.5	**48.4**	30.6	37.5

Table 3: Table presenting the absolute numbers and percentages of the Cuffcompare evaluation of the exon and transcript level for the single method predictions and the IPred, EVidenceModeler and Cuffmerge combinations on the simulated human data set. Note that only missed and novel exons are reported by Cuffcompare, but not the numbers for the transcript level. All IPred combinations are formed with AUGUSTUS predictions. The best values for each category are marked in bold. Abbreviations: Sn = sensitivity, Sp = specificity, F = Fmeasure.

4.3 *E.Coli* real data set

Table 4 shows the absolute values yielded by the Cuffcompare evaluation of the gene finders and gene prediction combinations while in Figure 3 a more detailed comparison of IPred combinations is presented. Note that we excluded the Cuffmerge results from the figure to achieve better visibility.
We see that including predictions only indicated by one or more evidence based gene finders results in an increase in sensitivity but also with a loss in specificity. Especially the "Glimmer3+GIIRA+nov" combination shows far more novel exons than the combination

excluding novel GIIRA predictions. This is as expected, since GIIRA is the method yielding the highest number of novel exons. Hence, IPred can filter out the novel predictions when only regarding identifications overlapped between different methods.
However, although including non-overlapping evidence based predictions reduces the specificity, it is still more specific than all other prediction methods, including EVidenceModeler or Cuffmerge. Further, also the overall accuracy of IPred combinations is improved compared to other combination methods and the single method predictions.

E.Coli real data set

method	missed	novel	sensitivity	specificity	Fmeasure
Glimmer3	59	1692	**93.9**	57.2	71.1
Cufflinks	704	188	5.2	7.9	6.3
GIIRA	190	4679	79.2	28.7	42.1
IPred_Cufflinks	796	**97**	65.8	**90.5**	76.2
IPred_Cufflinks+novel	709	154	66.4	78.4	71.9
IPred_GIIRA	279	338	84.7	83.0	83.8
IPred_GIIRA+novel	227	1189	88.3	62.8	73.4
IPred_Cufflinks+GIIRA	197	362	87.9	82.6	**85.2**
IPred_Cufflinks+GIIRA+novel	151	556	90.4	72.6	80.5
EVidenceModeler	123	1554	77.6	52.9	62.9
Cuffmerge	**11**	2808	10.4	6.7	8.1

Table 4: Table presenting the absolute numbers and percentages of the Cuffcompare evaluation for the *E.Coli* real data set. The best values for each category are marked in bold.

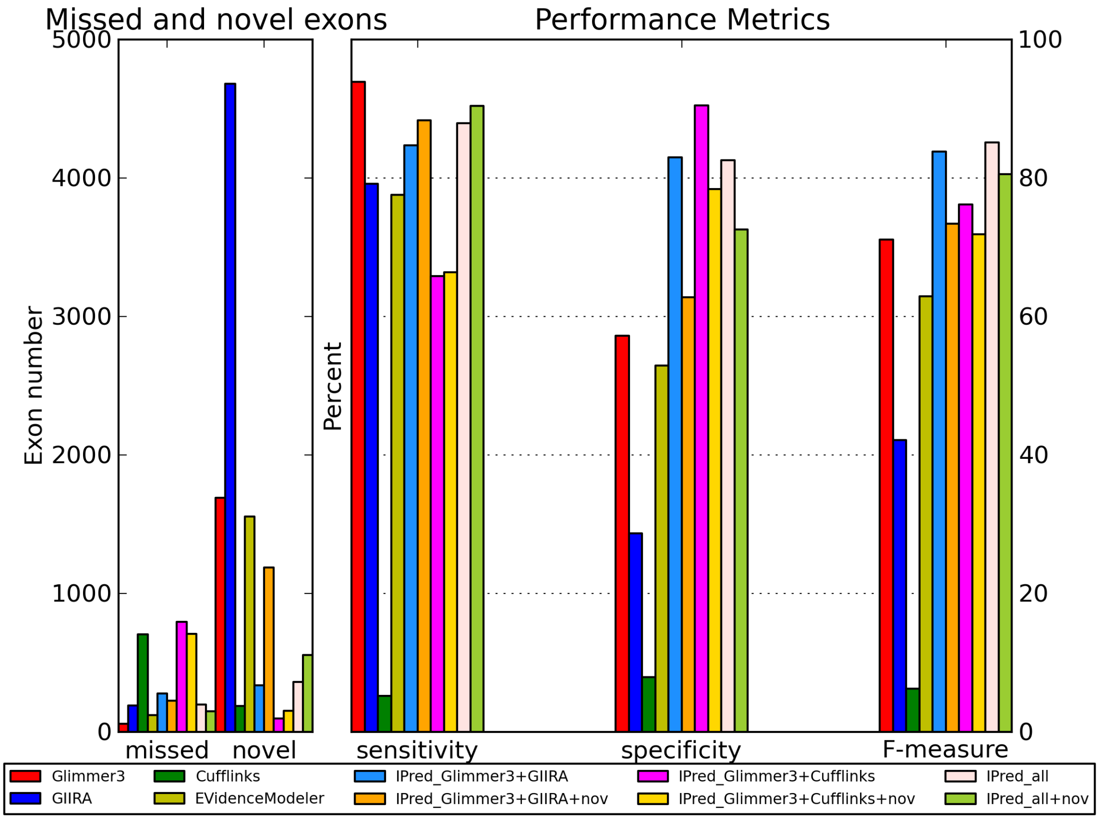

Figure 3: Overview of Cuffcompare metrics for the predictions of single methods and the method combinations for the *E.Coli* real data set. Note that "+nov" indicates that this combination not only reports the genes supported by all combined methods but also novel ones that have been predicted by the used evidence based methods.

4.4 Human real data set

Table 5 shows the absolute values for each compared Cuffcompare metric to accompany the analysis in the main manuscript.

Human real data set

			Exon			Transcript		
method	missed	novel	Sn	Sp	F	Sn	Sp	F
AUGUSTUS	13212	44753	**86.3**	74.3	**79.9**	**29.0**	25.0	26.9
GIIRA	52160	78092	43.6	38.1	40.7	0.0	0.0	0.0
Cufflinks	36061	49702	63.4	58.7	61.0	0.0	0.0	0.0
IPred_Cufflinks	38053	15255	74.3	**85.7**	79.6	25.6	**40.2**	**31.3**
IPred_Cufflinks+nov	33159	53719	75.9	65.3	70.2	25.6	11.9	16.2
IPred_GIIRA	69753	**14800**	58.4	83.7	68.8	22.4	38.4	28.3
IPred_GIIRA+nov	35019	86664	68.7	47.8	56.4	22.4	4.4	7.4
IPred_Cufflinks+GIIRA	41384	18308	72.1	83.0	77.2	24.5	33.8	28.4
IPred_Cufflinks+GIIRA+nov	39733	29112	72.5	77.1	74.7	24.5	21.1	22.7
EVidenceModeler	36134	37725	74.2	75.1	74.6	17.8	16.4	17.1
Cuffmerge	**10896**	114470	78.9	45.2	57.5	1.2	0.3	0.5

Table 5: Table presenting the absolute numbers and percentages of the Cuffcompare evaluation of the exon and transcript level for the single method predictions and the IPred, EVidenceModeler and Cuffmerge combinations on the human real data set. Note that only missed and novel exons are reported by Cuffcompare, but not the numbers for the transcript level. All IPred combinations are formed with AUGUSTUS predictions. The best values for each category are marked in bold. Abbreviations: Sn = sensitivity, Sp = specificity, F = Fmeasure.

4.5 EVidenceModeler - different weight settings

On each dataset we performed two runs with EVidenceModeler, one with equal weights for all methods, and one with higher weights assigned to methods based on evidence, as recommended on the EVidenceModeler webpage. Tables 6 and 7 present the Cuffcompare metrics for the two runs of each experiment.
As shown in the tables, the EVidenceModeler predictions using equal weights have a slightly better accuracy than using unequal weights. Hence, we chose to include the results based on equal weights in the comparison with IPred.

(1) *E.Coli* simulation - GeneMark based

weights	missed	novel	sensitivity	specificity	Fmeasure
equal	118	1039	81.7	65.3	72.6
unequal	155	1088	81.0	64.7	71.9

(2) *E.Coli* simulation - Glimmer3 based

weights	missed	novel	sensitivity	specificity	Fmeasure
equal	127	944	81.4	66.9	73.4
unequal	156	994	81.0	66.4	72.9

Table 6: Tables presenting the absolute numbers and percentages of the Cuffcompare evaluation of the exon level for the different weight settings of EVidenceModeler on the *E.Coli* datasets.

Human simulation

			Exon			Transcript		
weights	missed	novel	sensitivity	specificity	Fmeasure	sensitivity	specificity	Fmeasure
equal	6274	8753	79.5	76.0	77.7	33.3	27.2	29.9
unequal	6498	8923	78.6	75.1	76.8	30.0	25.0	27.3

Table 7: Table presenting the absolute numbers and percentages of the Cuffcompare evaluation of the exon and transcript level for the different weight settings of EVidenceModeler on the human dataset. Note that only missed and novel exons are reported by Cuffcompare, not the numbers for the transcript level.

4.6 Influence of the overlap threshold

IPred accepts a gene as supported also when an overlapping prediction of another method does not match perfectly. The threshold for the length of an overlap to be counted as sufficient support is per default 80% of the length of the gene of interest and can also be set by the user. Further, for transcripts the number of exons that need to be matched by an overlapping transcript from a second prediction method is also per default restricted to be at least as high as 80% of the total number of exons that correspond to the transcripts. In the following we shortly investigate the influence of the threshold to show that IPred is robust to different threshold settings and not biased by low thresholds.

Tables 8 and 9 show the comparison between the default overlap and an overlap threshold of 50%.

In particular for the *E.Coli* dataset we see that the differences between results obtained with the two overlap thresholds are only small and that the observed trends are similar. As expected, with a smaller threshold the sensitivity for the combinations is slightly improved, while the specificity is slightly reduced. In the end, this leads to very similar F-measures.

For the human simulation, the influence of the overlap threshold is more pronounced. Again, we observe an increase in sensitivity and a decrease in specificity when reducing the threshold. On the exon level the impact on the sensitivity increase is far more pronounced than in the prokaryotic simulation since we see considerable increases in sensitivity of up to 20%. However, this effect does not carry on to the transcript level, where the increase in sensitivity is much smaller and also coupled with a significant loss in specificity (6.5% to 9.3%). Hence, the strong effect on the exon level can be explained by the fact that AUGUSTUS exon chains often include an additional exon such that Cufflinks and GIIRA predictions does not match the prediction of AUGUSTUS. Reducing the overlap threshold hence results in more matches since more unequal exon numbers are accepted.

All in all, IPred is robust to the choice of the overlap threshold for prokaryotes and also for eukaryotes on the transcript level.

(1) *E.Coli* simulation - GeneMark based

threshold	combination with..	missed	novel	sensitivity	specificity	Fmeasure
50	Cufflinks	105	11	94.8	97.9	96.3
80		113	8	94.6	98.1	96.3
50	GIIRA	78	31	95.2	96.9	96.0
80		133	21	92.9	97.2	95.0
50	Cufflinks+GIIRA	55	30	96.2	97.0	96.6
80		58	20	96.1	97.3	96.7

(2) *E.Coli* simulation - Glimmer3 based

threshold	combination with..	missed	novel	sensitivity	specificity	Fmeasure
50	Cufflinks	136	21	92.3	96.2	94.2
80		142	12	92.2	96.5	94.3
50	GIIRA	107	37	93.1	95.4	94.2
80		163	20	90.8	96.0	93.3
50	Cufflinks+GIIRA	85	38	94.0	95.4	94.7
80		88	20	93.9	96.1	94.9

Table 8: Tables presenting the comparison between an overlap threshold of 80% and 50% for the *E.Coli* simulation.

Human simulation

				Exon			Transcript		
threshold	combination with..	missed	novel	Sn	Sp	F	Sn	Sp	F
50	Cufflinks	5619	1428	80.7	92.0	86.0	36.3	52.8	43.0
80		13921	600	60.5	95.1	74.0	33.1	61.0	42.9
50	GIIRA	5256	1641	81.4	91.4	86.1	36.7	51.6	42.9
80		13977	640	60.3	94.9	73.7	33.4	61.1	43.2
50	Cufflinks+GIIRA	9963	542	70.4	95.2	80.9	40.6	58.1	47.8
80		13792	275	61.3	96.9	75.1	37.8	64.6	47.7

Table 9: Table presenting the comparison between an overlap threshold of 80% and 50% for the human simulation. Note that only missed and novel exons are reported by Cuffcompare, but not the numbers for the transcript level. All IPred combinations are based on AUGUSTUS predictions. Abbreviations: Sn = sensitivity, Sp = specificity, F = F-measure.

References

Besemer, J., Lomsadze, A., and Borodovsky, M. (2001). GeneMarkS: a self-training method for prediction of gene starts in microbial genomes. Implications for finding sequence motifs in regulatory regions. *Nucleic Acids Res*, **29**(12), 2607–2618.

Burset, M. and Guigó, R. (1996). Evaluation of gene structure prediction programs. *Genomics*, **34**(3), 353–367.

CPLEX (2011). International Business Machines Corporation. v12.4: Users manual for CPLEX. *IBM ILOG CPLEX*.

Delcher, A. L., Bratke, K. A., Powers, E. C., and Salzberg, S. L. (2007). Identifying bacterial genes and endosymbiont DNA with Glimmer. *Bioinformatics*, **23**(6), 673–679.

Haas, B. J., Salzberg, S. L., Zhu, W., Pertea, M., Allen, J. E., Orvis, J., White, O., Buell, C. R., and Wortman, J. R. (2008). Automated eukaryotic gene structure annotation using EVidenceModeler and the Program to Assemble Spliced Alignments. *Genome biology*, **9**(1), R7.

Holtgrewe, M. (2010). Mason - a read simulator for second generation sequencing data. Technical Report TR-B-10-06, Fachbereich für Mathematik und Informatik, Freie Universität Berlin.

Kim, D., Pertea, G., Trapnell, C., Pimentel, H., Kelley, R., and Salzberg, S. (2013). TopHat2: accurate alignment of transcriptomes in the presence of insertions, deletions and gene fusions. *Genome Biol*, **14**(4), R36.

Langmead, B. and Salzberg, S. L. (2012). Fast gapped-read alignment with bowtie 2. *Nature methods*, **9**(4), 357–359.

Li, H., Handsaker, B., Wysoker, A., Fennell, T., Ruan, J., Homer, N., Marth, G., Abecasis, G., Durbin, R., and Subgroup, . G. P. D. P. (2009). The Sequence Alignment/Map format and SAMtools. *Bioinformatics*, **25**(16), 2078–2079.

Stanke, M., Schöffmann, O., Morgenstern, B., and Waack, S. (2006). Gene prediction in eukaryotes with a generalized hidden Markov model that uses hints from external sources. *BMC Bioinformatics*, **7**, 62.

Trapnell, C., Williams, B. A., Pertea, G., Mortazavi, A., Kwan, G., van Baren, M. J., Salzberg, S. L., Wold, B. J., and Pachter, L. (2010). Transcript assembly and quantification by RNA-Seq reveals unannotated transcripts and isoform switching during cell differentiation. *Nat Biotechnol*, **28**(5), 511–515.

Trapnell, C., Roberts, A., Goff, L., Pertea, G., Kim, D., Kelley, D. R., Pimentel, H., Salzberg, S. L., Rinn, J. L., and Pachter, L. (2012). Differential gene and transcript expression analysis of RNA-seq experiments with TopHat and Cufflinks. *Nat Protoc*, **7**(3), 562–578.

van Rijsbergen, C. J. (1979). Information retrieval. *London: Butterworths*, **2nd ed.**

Zickmann, F., Lindner, M. S., and Renard, B. Y. (2014). GIIRA – RNA-Seq driven gene finding incorporating ambiguous reads. *Bioinformatics*, **30**(5), 606–613.
